# The role of transoral fine needle aspiration in expediting diagnosis and reducing risk in head and neck cancer patients in the coronavirus disease 2019 (COVID-19) era: a single-institution experience

**DOI:** 10.1017/S0022215120001929

**Published:** 2020-09-02

**Authors:** P Touska, G Oikonomou, R Ngu, A Chandra, A Malhotra, A Fry, R Oakley, A Arora, J-P Jeannon, R Simo

**Affiliations:** 1 Department of Radiology, Guy's and St Thomas’ Hospitals NHS Foundation Trust, London, UK; 2 Department of ENT Surgery, Guy's and St Thomas’ Hospitals NHS Foundation Trust, London, UK; 3 Department of Dental Maxillofacial Imaging, Guy's and St Thomas’ Hospitals NHS Foundation Trust, London, UK; 4 Department of Cellular Pathology, Guy's and St Thomas’ Hospitals NHS Foundation Trust, London, UK

**Keywords:** Oropharyngeal Cancer, Transoral, Fine-Needle Aspiration, Cytology, Coronovirus

## Abstract

**Objective:**

The global coronavirus disease 2019 (COVID-19) pandemic has necessitated rapid alterations to diagnostic pathways for head and neck cancer patients that aim to reduce risk to patients (exposure to the hospital environment) and staff (aerosol-generating procedures). Transoral fine needle aspiration cytology offers a low-risk means of rapidly diagnosing patients with oral cavity or oropharyngeal lesions. The technique was utilised in selected patients at our institution during the pandemic. The outcomes are considered in this study.

**Method:**

Diagnostic outcomes were retrospectively evaluated for a series of patients undergoing transoral fine needle aspiration cytology of oral cavity and oropharyngeal lesions during the COVID-19 pandemic.

**Results:**

Five patients underwent transoral fine needle aspiration cytology, yielding lesional material in 100 per cent, with cell blocks providing additional information. In one case, excision biopsy of a lymphoproliferative lesion was required for final diagnosis.

**Conclusion:**

Transoral fine needle aspiration cytology can provide rapid diagnosis in patients with oral cavity and oropharyngeal lesions. Whilst limitations exist (including tolerability and lesion location), the technique offers significant advantages pertinent to the COVID-19 era, and could be employed in the future to obviate diagnostic surgery in selected patients.

## Introduction

The global coronavirus disease 2019 (COVID-19) pandemic had, as of 23rd June 2020, resulted in over 8.8 million confirmed cases and almost 0.5 million deaths worldwide.^1^ Severe acute respiratory syndrome coronavirus-2 (SARS-CoV-2) infection results in a severe or critical illness in 20–30 per cent of cases and has a fatality rate of 1.4–7.2 per cent.^2^ However, the disease affects individuals differently; in particular, it disproportionately affects the elderly (with a case fatality rate of 8.0–22.5 per cent for those aged over 70 years) and individuals with co-morbid conditions.^3^ This finding is relevant to head and neck cancer patients, many of whom fall into this higher risk group.

Prior to the onset of the pandemic, surgical procedures such as diagnostic panendoscopy and surgical biopsy were the standard of care; these procedures were integral to the diagnosis and management of oral cavity, oropharyngeal, laryngeal and hypopharyngeal cancers.^4,5^ However, emerging data following the spread of Covid-19 suggest that head and neck cancer surgical procedures may entail a higher risk to patients and staff.^6,7^

Novel barriers to safe head and neck cancer surgery include imperfect pre-surgical screening for Covid-19, as there is an elevated risk to patients of developing severe respiratory illness post-operatively (secondary to infection by the virus) and to staff (owing to prolonged SARS-CoV-2 aerosolisation during lengthy, or multiple, aerosol-generating procedures (AGPs)). Additionally, enhanced personal protective equipment (PPE) may be incompatible with routine operative equipment and its supply may be limited.^8^ Therefore, at our institution, there has been a focus on providing a diagnosis via an approach least likely to require hospital admission, an AGP (including intubation) or repeat attendance at hospital.

Transmucosal transoral fine needle aspiration cytology (FNAC) is a well-established diagnostic technique. It offers a low-morbidity, minimally invasive means of obtaining a cytological diagnosis for oropharyngeal and oral cavity lesions.^9^ Its role has been documented in the literature, with significant advantages in specific cases.^10^ In particular, it can be performed on an out-patient basis, minimising patients’ exposure to the hospital environment and exposure of staff to a more lengthy surgical AGP. Furthermore, material obtained via transoral FNAC can be subjected to immunohistochemical staining or in situ hybridisation (applied to a cell block), obviating the need for an incisional or excisional biopsy in some cases.^11^

This paper presents a series of consecutive cases where transoral FNAC was used to establish a diagnosis in patients with oropharyngeal or oral cavity lesions during, and in the lead-up to, the COVID-19 related lockdown in the UK.

## Materials and methods

Five patients underwent transoral FNAC during, and in the lead-up to, the Covid-19 related lockdown in the UK. The diagnostic protocol was already in place as part of the standard operational policy of the multidisciplinary team (MDT) head and neck tumour board of our unit. No ethical approval was required for the study, as it was part of the current research and development guidance of our institution.

### Patient selection

Patient selection for transoral FNAC was carried out using a multidisciplinary approach. In all cases, transoral FNAC was considered, by both clinical and diagnostic specialists, to be capable of providing additive or sufficient information to guide further management and expedite definitive treatment. The target lesions were also confirmed to be amenable to transoral FNAC, based upon a number of considerations, including clinical factors, lesion location and radiological information (Table 1).^12^

**Table 1.** Criteria for transoral FNAC feasibility

Patient factors
– Able to consent, & sit or lie still for procedure
– Sufficient mouth opening & lack of hypersensitive gag reflex
– No allergies to topical or local anaesthesia
Coagulation
– FBC, INR, platelet count available & within suitable range (as per Society of Interventional Radiology Consensus guidelines).^16^ NB Thresholds are more permissive than for surgical intervention, as FNA of such lesions is deemed a low bleeding risk procedure
– Concurrent use of anticoagulants should be noted; withholding should be considered, but may not be necessary (as per Society of Interventional Radiology Consensus guidelines)^16^
Lesion location
– Oral cavity
– Oropharynx accessible via oral cavity approach (lesions lying below level of upper tongue base & those arising from posterior pharyngeal wall are unlikely to be accessible)
– Parapharyngeal or retropharyngeal (typically only when large & with ultrasound guidance)
Radiological factors
– Absence of significant overlying vessels
– Sampling path will not risk injury to significant neural structures
– No features to suggest highly vascularised lesion likely to bleed significantly on sampling
Operational factors
– Transoral FNAC is rapidly available & will not delay diagnosis (even in cases of non-diagnostic sampling)
– Rapid ENT or airway specialist support available within hospital in event of complications

FNAC **=** fine needle aspiration cytology; FBC = full blood count; INR = international normalised ratio; FNA **=** fine needle aspiration

### Safety

In accordance with institutional and national guidance protocols established to manage the COVID-19 pandemic,^13^ patients were issued with facemasks and social distancing was maintained whilst within the department. The procedures, and the donning and doffing of PPE, were carried out in rooms within the radiology department designated as safe for the conduct of AGPs on patients who could be infected with COVID-19. All staff within the procedure room, including a radiologist, radiology department assistant and cytology biomedical scientist, were issued with filtering facepiece code 3 (FFP3) masks, disposable gowns, aprons and visors. A designated ‘runner’ was available to pass the prepared slides to a cytopathologist, located in a separate adjacent room.

### Transoral fine needle aspiration cytology procedure

**Fig. 1.**
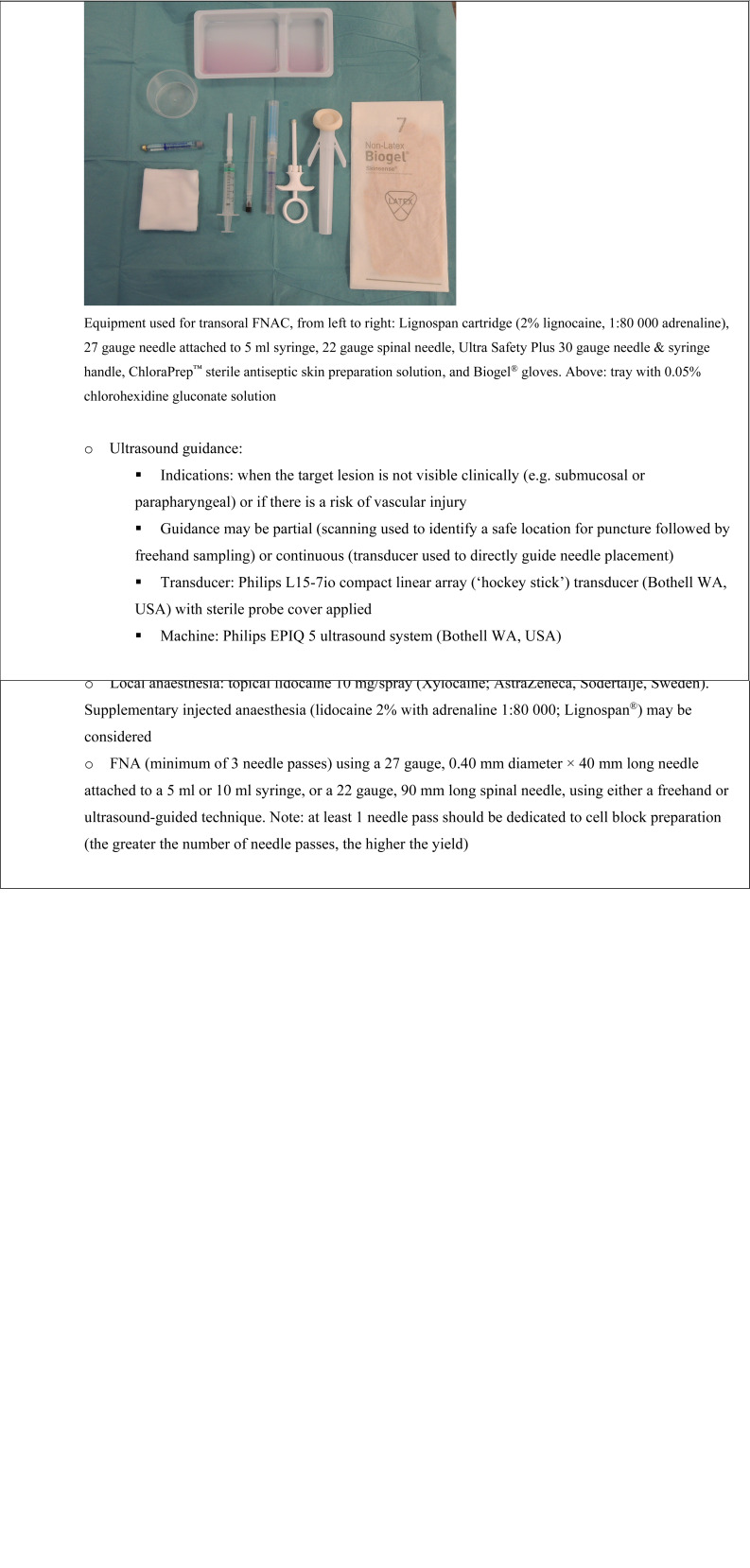
Equipment and technique employed for the transoral fine needle aspiration (FNA) cytology (FNAC) procedure.

The procedural aspects of transoral FNAC are detailed in Figure 1.

### Diagnostic neck ultrasound

In each case, systematic scanning of the neck soft tissues was carried out by the same radiologist operator with a Philips eL18-4 linear transducer (Bothell, Washington, USA) using a standardised multiple ‘sweep’ technique.^14^ This enables identification (and FNAC or core biopsy if required) of pathological cervical lymph nodes for disease staging purposes.

### Rapid onsite cytology

Air-dried and ethanol-fixed direct spread preparations were made by a cytology biomedical scientist located within the procedure room. The air-dried preparations were stained onsite with Hemacolor (Merck, Darmstadt, Germany) whilst the ethanol-fixed smear was stained with a Papanicolaou stain later in the lab. The Hemacolor stained slides were reviewed onsite by a cytopathologist located in an adjacent room using an Olympus BX50 microscope (Tokyo, Japan).

Rapid onsite evaluation is standard practice at our institution; therefore, the resources and expertise were available for us to consider adaptation to performing transoral fine needle aspiration (FNA) during the COVID-19 pandemic. In each case, a provisional diagnosis is provided to the radiologist, together with recommendations on performing further needle passes if necessary. Needle washings may also be obtained and triaged depending on the cytomorphological evaluation of direct smears (e.g. for cell block preparation, flow cytometry or microbiology). Following cell block preparation, haematoxylin and eosin stained sections can be examined, and special or immunohistochemical stains performed, as appropriate.

### Data collection

Information collected included age, medical co-morbidities, primary lesion location, cytological analysis results, core biopsy findings (if performed), associated neck ultrasound staging findings, final histopathological diagnosis (if available), disease staging and management. Searches were performed using electronic patient records and the picture archiving and communication system.

## Results

A total of five patients underwent transoral FNAC during (*n* = 4) and in the immediate lead-up (*n* = 1) to the COVID-19 related national lockdown in the UK. Four patients (80 per cent) had significant co-morbidities, and one patient (20 per cent) tested positive for Covid-19 (Table 2).

**Table 2.** Patient characteristics

Patient number	Age (years)	Co-morbidities	Covid-19 status (pre-procedure)
1	29	None	Unknown
2	62	Seizures, cardiac problems, gastrostomy dependent	Negative
3	75	Stroke. Unsuitable for general anaesthesia	Negative
4	37	Hypertension, diabetes, asthma	Unknown
5	62	Thrombocytopenia, immune deficiency	Positive

Covid-19 = coronavirus disease 2019

**Fig. 2.**
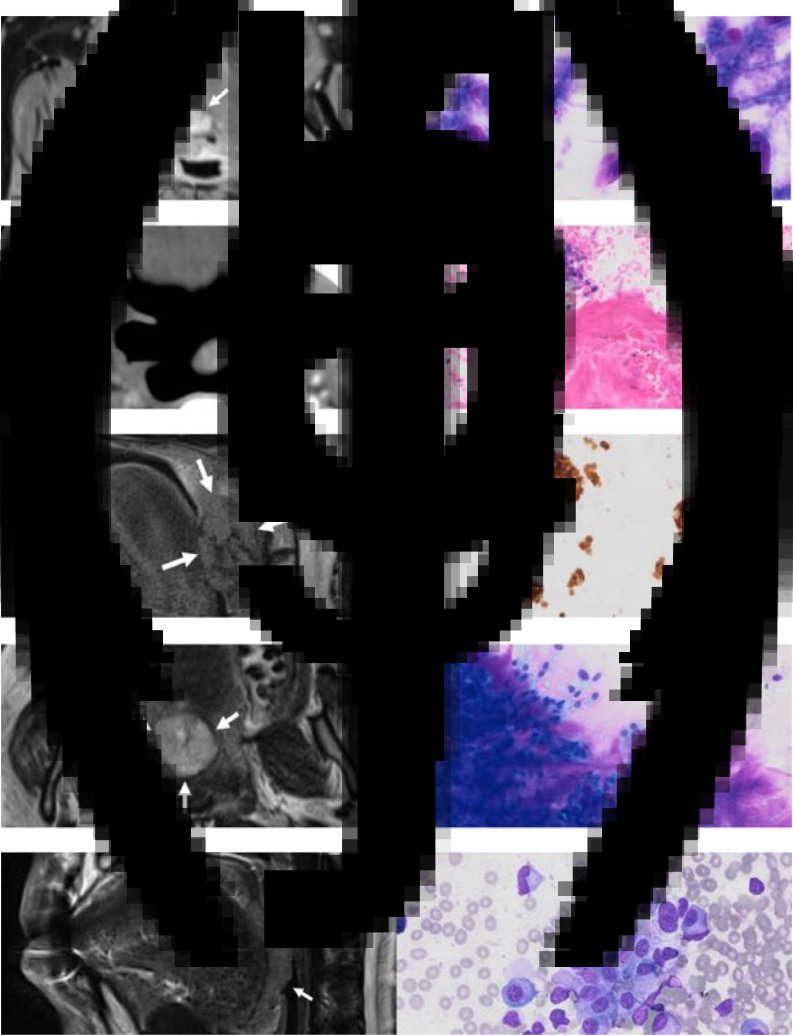
Imaging and cytology from the five cases. (a) Axial, fat-suppressed, post-contrast, Dixon T1-weighted magnetic resonance imaging (MRI) scan demonstrating an enhancing right-sided paramedian lesion at the superior aspect of the tongue base (arrows). (b) Direct smear (onsite) Hemacolor stain (×20 magnification) demonstrating cytological features of an adenoid cystic carcinoma. (c) Axial, post-contrast computed tomography scan demonstrating a left-sided enhancing oropharyngeal lesion involving the palatine tonsil and tonsillar pillars (arrows). (d) Cell block H&E stain (×10 magnification) demonstrating a poorly differentiated squamous cell carcinoma (SCC) (p16 negative) with keratinisation and a small group of pleomorphic malignant cells. (e) Sagittal, T2-weighted MRI scan demonstrating a bulky right-sided palatine tonsillar mass (arrows). (f) Cell block p16 immunostain (×10 magnification) demonstrating features of a non-keratinising, poorly differentiated SCC. (g) Axial, T2-weighted MRI scan demonstrating a rounded right-sided paramedian palatal mass (arrows). (h) Direct smear (onsite) Hemacolor stain (×20 magnification) demonstrating features of a pleomorphic adenoma. (i) Sagittal, T2-weighted MRI scan demonstrating an exophytic left-sided tongue base mass (arrows). (j) Direct smear Hemacolor (onsite) stain (×20 magnification) demonstrating a plasma cell infiltrate.

All patients underwent pre-procedural cross-sectional imaging (Figure 2). All patients were deemed amenable and suitable for transoral FNAC based upon clinical and radiological factors. All underwent contemporaneous cervical ultrasound for staging purposes. There were no procedure-related complications (Table 3).

**Table 3.** Imaging and procedural complications

Patient number	Primary lesion location	Cross-sectional imaging	High-risk features?	Diagnostic ultrasound neck findings	Needle passes (*n*)	Complications
1	Junction of oral tongue & tongue base (right)	MRI	No	No cervical lymphadenopathy	5	None
2	Palatine tonsil (left)	MRI + CT	No	Bilateral suspicious level 2A nodes subject to FNA (ipsilateral positive for malignancy, contralateral negative)	3	None
3	Palatine tonsil (right)	MRI + CT	No	Enlarged suspicious ipsilateral level 1B node subject to FNA (negative for malignancy)	4	None
4	Junction of hard & soft palate (right)	MRI	No	No cervical lymphadenopathy	2	None
5	Tongue base (left)	MRI	No	No cervical lymphadenopathy	3	None

MRI = magnetic resonance imaging; CT = computed tomography; FNA = fine needle aspiration

Lesional material could be obtained in all cases (100 per cent) and was sufficient for diagnosis in 80 per cent of cases (*n* = 4). In 20 per cent of cases (*n* = 1), the correct diagnosis was suggested on cytology, but excision biopsy was required for the final diagnosis of the lymphoproliferative lesion (Table 4). Material for cell block was obtained in 60 per cent of cases (*n* = 3); of these, one cell block was acellular (although it did not influence the cytological diagnosis), one was sufficient for immunohistochemical staining (including P16) and one was sufficient for limited immunohistochemical staining, which provided support for the cytomorphological diagnosis.

**Table 4.** Cytology and histology

Patient number	Lesional material obtained?	Cytological diagnosis	Cell block & IHC	Cytology sufficient for diagnosis?	Histology
1	Yes	Adenoid cystic carcinoma	Acellular, but not required for diagnosis	Yes	Adenoid cystic carcinoma
2	Yes	Poorly differentiated SCC	Not required (contemporaneous ultrasound-guided nodal core biopsy)	Yes	Poorly differentiated SCC, p16 negative (based on ultrasound-guided core biopsy of necrotic node, carried out at time of transoral FNAC)
3	Yes	Poorly differentiated non-keratinising SCC	p16 positive	Yes	–
4	Yes	Pleomorphic adenoma	Not required for diagnosis	Yes	–
5	Yes	Suspected PTLD	Lymphoid cells of mixed CD20+/CD79a+ B-cells & CD3+ T-cells. Plasma cells highlighted by CD79a & MUM1, with kappa light chain restriction. In situ hybridisation for EBER & IHC findings for HHV8 are negative	No (histology is ‘gold standard’ for PTLD diagnosis)	PTLD (polymorphic type)

IHC = immunohistochemistry; SCC = squamous cell carcinoma; FNAC **=** fine needle aspiration cytology; PTLD = post-transplant lymphoproliferative disorder; CD = cluster of differentiation; MUM1 = multiple myeloma oncogene 1; EBER = Epstein–Barr virus encoded RNA; HHV8 = human herpesvirus type 8

The key imaging and cytological features of the five lesions subjected to transoral FNAC are provided in Figure 2. The final diagnosis, staging and treatment for each case are summarised in Table 5.

**Table 5.** Staging and management

Patient number	Final diagnosis	TNM staging	Treatment
1	Adenoid cystic carcinoma	T_1_N_0_M_0_	Transoral robotic partial glossectomy, neck dissection & post-operative radiotherapy
2	Poorly differentiated SCC	T_2_N_2b_M_0_	Radical radiotherapy
3	Poorly differentiated non-keratinising SCC	T_4_N_0_M_0_	Palliative radiotherapy
4	Pleomorphic adenoma	–	Surgical resection
5	Post-transplant lymphoproliferative disorder (polymorphic type)	–	Patient referred to haematology for management

TNM = tumour–node–metastasis; SCC = squamous cell carcinoma

## Discussion

The COVID-19 pandemic has resulted in rapid and radical reconsiderations of both the diagnosis and treatment of patients with suspected or confirmed head and neck cancer. With regard to diagnosis, the drive has primarily been towards minimising patient exposure to the hospital environment (including avoiding multiple or lengthy attendances at hospital) as well as minimising staff exposure to AGPs.^15,16^ Despite this, the diagnosis of suspected upper aerodigestive tract cancers typically requires a surgical biopsy, which entails risk not only to the patient (from general anaesthesia and a longer period of exposure to the hospital environment), but also to clinicians (as a consequence of aerosol-generating intubation and upper aerodigestive tract instrumentation).

Transoral FNAC offers a means of achieving a rapid (cytological) diagnosis for lesions of the oral cavity, oropharynx and parapharyngeal space, without the need for general anaesthesia. It is also ideally suited to one-stop and clinic settings (with appropriate cytopathology support). Furthermore, coagulation thresholds are typically more permissive for FNA when compared to incisional or excisional biopsies, enabling the technique to be offered to a wider range of patients.^12^

Whilst the use of transoral FNAC is not widespread in the UK, it was occasionally employed at our institution. Its use is also well documented in the literature, with high levels of sensitivity and specificity reported.^17–25^ Indeed, Deng *et al*. used intra-oral FNA in 28 patients with benign and malignant oral and oropharyngeal lesions. The authors recorded sensitivity, specificity and accuracy values of 100 per cent, 95 per cent and 97 per cent, respectively.^23^

However, there are limitations to the technique, including tolerability (the procedure may be impossible in patients with trismus or a sensitive gag reflex), and not all lesions are accessible transorally (such as those within the nasopharynx, inferior oropharynx, hypopharynx and larynx). Furthermore, obtaining aspirates from lesions within the parapharyngeal space often requires correlation with cross-sectional imaging and/or direct ultrasound guidance to ensure safe practice.^26^ Finally, obtaining sufficient material for diagnosis can be challenging, as patients may not tolerate multiple needle passes. This is particularly pertinent where additional information (beyond cytomorphology alone) is required, such as human papillomavirus (HPV) status in oropharyngeal squamous cell carcinoma cases, which requires P16 immunohistochemical staining of a cell block.^22,23,27^

Prior to the onset of the COVID-19 pandemic, patients with possible or suspected head and neck cancer at our institution would typically need to attend the hospital for clinical assessment, imaging, diagnostic biopsy of the primary lesion and panendoscopy (under general anaesthesia), before definitive treatment could be decided upon. However, following the onset of the Covid-19 pandemic, our institution introduced changes in line with recommendations and guidelines produced by national organisations, such as ENT UK and the British Association of Head and Neck Oncologists.^15,16^ Changes included the introduction of telephone-based virtual clinics, aided by published risk assessment tools.^16^ In addition, there was an effort to reduce both the number and length of hospital attendances, as well as minimise AGPs.

As part of the effort to minimise risks to patients and staff, transoral FNAC (which was already employed in select cases) was utilised to provide a diagnosis in patients with suspected head and neck cancer at our institution, where appropriate. All cases in our series were discussed at an MDT tumour board meeting to ensure appropriate patient selection. In particular, all lesions were located within the oral cavity or oropharynx, and where lesions involved the tongue base, there was sufficient superior extension to enable a transoral approach. Being cognisant of the lower risks of FNA, as compared to a surgical biopsy performed under general anaesthesia, co-morbidities were also considered. In our series, 80 per cent of patients had significant co-morbidities, including one patient who was deemed unfit for general anaesthesia and one patient with thrombocytopenia (platelet count of 55 × 10^9^/l on the day of the procedure).

Overall, transoral FNAC yielded lesional material in 100 per cent of cases in our series and provided a definitive diagnosis in 80 per cent (*n* = 4). In 20 per cent of cases (*n* = 1), post-transplant lymphoproliferative disorder was suspected on the transoral FNAC specimen, but an excision biopsy was required to make the final diagnosis (owing to the need to assess tissue architecture and carry out extensive ancillary studies). Although histology is the ‘gold standard’ for the diagnosis of post-transplant lymphoproliferative disorder, transoral FNAC was clinically useful in this case, as the specimen was negative for a primary epithelial malignancy, enabling rapid triage to an appropriate clinical specialty (haematology) for further management.

In each case, the provision of a preliminary diagnosis (and adequacy of individual needle passes) was possible immediately after sampling, prompting further needle passes (if appropriate) and more rapid clinical decision making. This contrasts with results from an excisional biopsy, which typically take longer to process. Nevertheless, additional time is required where cell block processing and immunohistochemical staining are required. In our series, a cell block was prepared in three cases (60 per cent), enabling P16 staining (for high-risk HPV status) and support for the provisional diagnosis of post-transplant lymphoproliferative disorder.

A further advantage of transoral FNAC carried out by a radiologist capable of diagnostic ultrasound scanning of the neck was the ability to carry out comprehensive nodal staging during a single hospital attendance. In one case, a concurrent core biopsy of a cervical lymph node enabled P16 staining to be achieved, obviating the need for cell block preparation or a surgical biopsy.

- Transoral fine needle aspiration cytology (FNAC) offers a low-risk means of rapidly diagnosing patients with oral cavity or oropharyngeal lesions
- Transoral FNAC has proven useful during the COVID-19 pandemic
- It can reduce risks to patients and staff associated with repeated hospital attendances and prolonged aerosol-generating procedures
- Lesion location and procedure tolerability are important when deciding whether transoral FNAC may be beneficial; radiological input is essential
- Onsite cytopathology expertise enables assessment of material for adequacy and rapid diagnosis
- Cytological specimens can be used to create cell blocks for immunohistochemical staining (e.g. to confirm human papillomavirus status)

In cases (such as the latter) where tissue can be obtained from nodal metastases and the associated primary head and neck cancer is clear based on clinical and radiological grounds, one might question whether obtaining tissue from the primary lesion is necessary. In our institution, only patients in whom palliative treatment is being considered would not proceed to sampling of the primary lesion; however, limitations imposed by the COVID-19 pandemic may lead to greater acceptance of this approach.

### Limitations

The authors acknowledge the limitations of this study, including the small sample size, which is predominantly a result of the need for rigorous patient selection, particularly with respect to lesion location (as discussed above). It is also important to note the limitations of cytology in obtaining a definitive diagnosis in all cases. In particular, there has been debate about the reliability and cut-offs for P16 staining of cell block material in the setting of head and neck squamous cell carcinoma, and in some laboratories liquid-based HPV DNA testing has been utilised.^11^

### Added value

The current paper highlights the utility of transoral FNAC in diagnosing oral cavity and oropharyngeal lesions in patients with suspected head and neck cancer pertinent to the COVID-19 era. In particular, it demonstrates that a definitive diagnosis may be made in the majority of cases, obviating the need for surgical biopsy and its associated risks. As a result, it offers a potentially game-changing tool in the diagnostic armamentarium of clinicians investigating patients with suspected head and neck cancer, when appropriate patient selection is applied.

## Conclusion

The onset of the COVID-19 pandemic has caused departments to re-evaluate their pathways and seek means of minimising risk. Transoral FNAC, with rapid onsite assessment by a cytopathologist, can obviate the need for surgical biopsy in selected head and neck cancer patients. This can in turn reduce risk to patients and staff pertinent to the COVID-19 era. However, limitations exist, including patient tolerability, lesion location and obtainment of sufficient material for immunohistochemistry. Patient selection and relevant multidisciplinary expertise are essential.
